# Telerehabilitation exercise program in pediatric patients after kidney transplantation: a randomized clinical trial

**DOI:** 10.1007/s00467-025-07123-3

**Published:** 2026-02-04

**Authors:** Raquel P. Carbonera, Amanda Alves Luft, Ana Clara Sobotyk, Karolayne de Lima Recoba, Clotilde Druck Garcia, Janice Luisa Lukrafka

**Affiliations:** 1 Graduate Program in Pediatrics: Child and Adolescent Care, Universidade Federal de Ciências da Saúde de Porto Alegre (UFCSPA), Porto Alegre, RS Brazil; 2 Undergraduate in Physical Therapy, Universidade Federal de Ciências da Saúde de Porto Alegre (UFCSPA), Porto Alegre, RS Brazil; 3 Graduate Program in Pediatrics: Child and Adolescent Care, Universidade Federal de Ciências da Saúde de Porto Alegre (UFCSPA) and Department of Pediatrics (UFCSPA), Santo Antonio Children Hospital – Santa Casa de Porto Alegre, Porto Alegre, RS Brazil; 4 Department of Physical Therapy, Graduate Program in Pediatrics: Child and Adolescent Care and Program in Rehabilitation Sciences, Janice Luisa Lukrafka, Universidade Federal de Ciências da Saúde de Porto Alegre (UFCSPA), Porto Alegre, RS Brazil

**Keywords:** Transplanted kidney, Chronic renal insufficiency, Child, Adolescent, Exercise, Telerehabilitation

## Abstract

**Background:**

Although kidney transplantation (KT) improves kidney function, children with chronic kidney disease (CKD) often show incomplete recovery of physical function. Physical activity is recommended, and telerehabilitation may enhance adherence. This study evaluated the effects of a telerehabilitation exercise program on functional capacity in pediatric KT recipients. Secondary outcomes included peripheral muscle strength, quality of life (QoL), laboratory measures, and program safety.

**Methods:**

This single-blind randomized clinical trial included two groups: intervention (IG) and control (CG). Functional capacity (Modified Shuttle Walk Test – MSWT), handgrip strength, and laboratory measures were assessed before and after the intervention. Both groups participated in remote sessions twice weekly for six weeks. The IG performed age-appropriate physical exercises, while the CG completed simple ventilatory exercises and a structured interview.

**Results:**

51 patients were randomized (IG = 25; CG = 26). There were no significant differences in baseline characteristics between groups. The IG showed a 90.6-m increase in MSWT distance, while the CG improved by only 0.8 m. Both groups demonstrated gains in peripheral muscle strength, with greater improvements in the IG. Laboratory measures showed no significant changes. The program was feasible and safe.

**Conclusion:**

Significant improvements in functional capacity were observed in pediatric KT patients. Although no statistically significant differences were observed in peripheral muscle strength or QoL between groups, both improved, especially in the IG. Laboratory parameters remained unchanged. The intervention was safe and demonstrated good adherence, highlighting its potential for post-transplant rehabilitation.

**Trial Registration:**

Brazilian Registry of Clinical Trials (ReBEC), RBR-5gm65y8.

**Graphical Abstract:**

**Figure:**
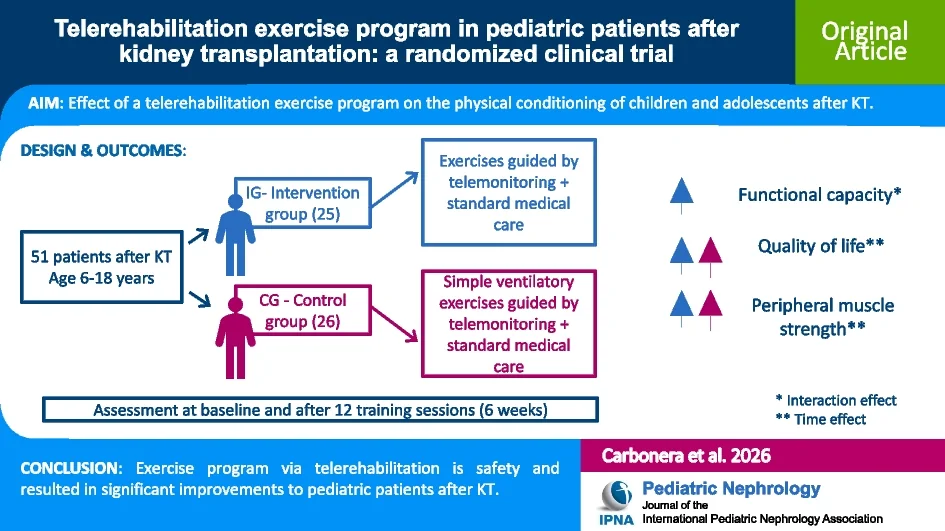
A higher resolution version of the Graphical abstract is available as Supplementary information

**Supplementary Information:**

The online version contains supplementary material available at https://doi.org/10.1007/s00467-025-07123-3.

## Introduction

Kidney transplantation (KT) is recognized as a crucial therapeutic option in patients with chronic kidney disease (CKD) stage 5, especially children. Preemptive transplantation is particularly recommended in this population to reduce complications such as growth impairment and renal osteodystrophy and significantly improve the patient's life expectancy and quality of life (QoL) [1–3].

Despite promising results in kidney function after transplantation, several aspects related to the functionality of children with CKD are not fully restored following the procedure [4–7]. Significant dysfunctions persist due to the systemic effects of circulating toxins, excess body volume, medication use, and anemia, which can continue to impact patients even after the procedure [6, 8]. This may impair respiratory and peripheral muscle strength, exercise capacity, and QoL in this population [5–7, 9].

Few studies have investigated the effect of structured exercise programs on children with CKD and those undergoing KT. It is well established that progressive resistance exercises conducted in person lead to significant improvements in both QoL and functional capacity in children with CKD [10]. Furthermore, Lubrano et al. [11] found that physical exercise performed 3 to 5 h per week significantly improved cardiorespiratory fitness and left ventricular mass in children following successful KT, suggesting that the resumption of appropriate exercise after KT should be strongly encouraged.

Telehealth is a technological tool that has improved child health globally. It enables hospital and outpatient care, educates professionals and patients, conducts research, responds to emergencies, and provides access to pediatric care in remote and underserved areas [12]. Telerehabilitation tools for implementing physical activity and exercise in pediatrics have yielded promising results, particularly in studies of chronic conditions such as asthma [13] and obesity [14]. The sustained benefits of these interventions over time — increased physical activity and improved fitness — have recently been documented [15].

A recent literature review highlights that telemedicine for patients after KT can offer benefits, including reduced travel time and costs, improved medication adherence, increased self-sufficiency, and more reliable blood pressure readings [16]. However, specific information on exercise programs and physical exercise after KT in the pediatric population, especially using telerehabilitation, has not yet been reported, and studies are needed to fill this gap. Therefore, this study aimed to evaluate the effect of a telerehabilitation exercise program on functional capacity in children and adolescents after KT. Second, we evaluated the peripheral muscle strength, QoL, laboratory measures, and safety of the exercise program.

## Materials and methods

### Patient population and study design

This single-blind randomized clinical trial was performed at the Pediatric Nephrology Outpatient Clinic at the Hospital da Criança Santo Antônio —Complexo Hospitalar Santa da Casa de Porto Alegre (Brazil) from August 2022 to August 2024. The Institutional Ethics Committee approved the protocol, and written consent was obtained from each child’s parents or relatives. The study was included in the Brazilian Registry of Clinical Trials (code: RBR-5gm65y8).

The sample comprised recent KT recipients (between 1 and 12 months after transplantation) of both sexes, aged between 6 and 18. Patients were excluded if they had underlying severe pulmonary or cardiologic disease, neurological disease, orthopedic disease, or cognitive impairment, which would limit their ability to understand the training protocol.

Following the recommendations of the *Consolidated Standards of Reporting Trial* (CONSORT) [17], patients were invited to participate. After signing the consent forms, children were randomly assigned to the intervention group (IG) or the control group (CG). A researcher (JLL) performed the randomization using a computerized random number generator (randomization.com) in blocks of four.

### Logistics and study procedures

A data collection form was used to gather demographic characteristics, including age, sex, height, and weight. Patients' weight and height were measured using a calibrated digital scale and a stadiometer. Information on medications used, length of stay in the intensive care unit (ICU), and participation in physical activities was also collected. This study defined valid physical activity as activities performed at least three times a week for a minimum of 30 min per day. All participants underwent the same protocol for assessing maximum functional capacity, peripheral muscle strength, and QoL, at the beginning of the study and after 12 training sessions. The assessments were conducted by trained researchers who were unaware of the participants' allocation.

Functional capacity was assessed using the *Modified Shuttle Walk Test* (MSWT). This test evaluates exercise capacity by measuring the maximum distance an individual can cover while following progressively increasing speeds dictated by an audio signal [18, 19]. Conducted on a 10-m circuit, the test consists of 15 levels and is terminated if the participant is unable to maintain the required pace due to dyspnea, fatigue, or repeated failure to complete a lap within the allotted time. At the end of the test, the total distance covered is measured. Oxygen saturation and heart rates were assessed at the beginning, at the end of the test, and after 5 min of rest using a pulse oximeter (Oled Graph G-tech, model OXIOLCM, Brazil), while blood pressure was measured with a digital sphygmomanometer (OMRON, model HEM-7130, Brazil) using appropriately sized cuffs, with the patient seated. Additionally, respiratory rates and Borg scores [20] were used to assess dyspnea and the intensity of leg fatigue during physical activity. Absolute values were evaluated according to the references described in Lanza's study [21].

Peripheral muscle strength was assessed using a JAMAR® hand dynamometer, with results reported in kilograms (kg). The test was conducted in accordance with the recommendations of the *American Society of Hand Therapists* [22], and participants received verbal encouragement to exert maximum effort during each measurement. The individual performed the maximum grip for 10 s and recorded the highest peak force. Three measurements were taken alternately with the dominant and non-dominant hands. The mean of the three measurements for each hand was calculated and compared with the reference values proposed by McQuiddy [23] for children and adolescents, stratified by age and sex.

Quality of life was evaluated using the standardized, validated Pediatric Quality of Life Inventory (PedsQL™) version 4.0, translated into Portuguese (Brazil). The questionnaire consists of 23 items divided into four modules (physical dimension, emotional, social, and school dimensions, with five items each). Children respond using a five-point scale (0 = never; 1 = almost never; 2 = sometimes; 3 = often; 4 = almost always a problem). The final total score indicates that higher scores are associated with better QoL [24]. Scores below 50% were considered poor QoL, between 50% and less than 75% fair QoL, and between 75 and 100% good QoL [25].

Laboratory measures were performed according to the patient's routine blood collection procedures. The variables assessed included hematocrit and hemoglobin levels, urea, creatinine, and serum potassium levels. Blood samples were collected at baseline and after 12 training sessions in both groups. The Schwartz formula [26] was used to estimate glomerular filtration rate (eGFR).

### Training program

**Fig. 1:**
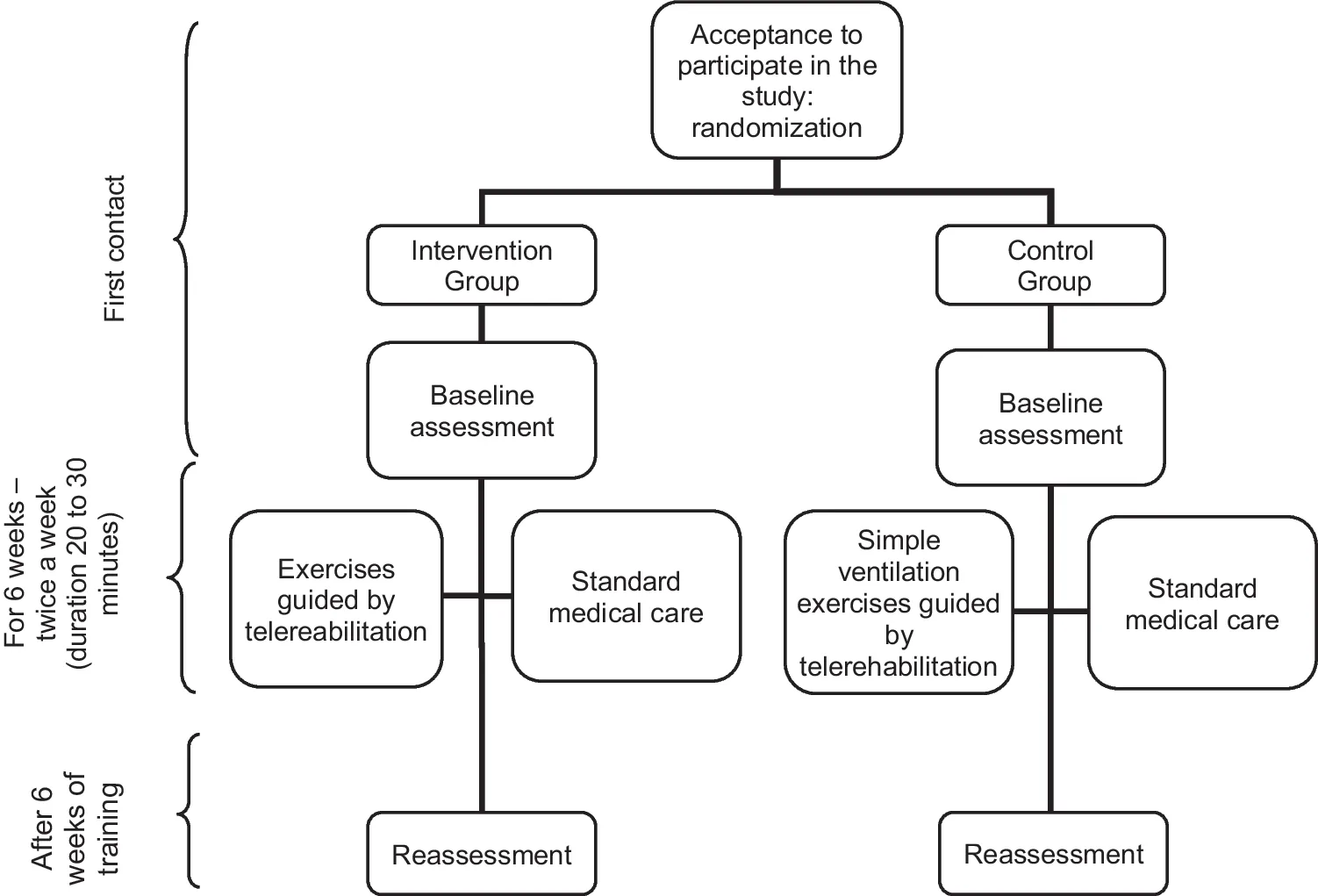
Flow chart of selection, interventions, and follow-up—reproduced from Carbonera et al. [28]

The telerehabilitation program was conducted twice a week for 6 weeks, comprising 12 sessions (Fig. 1), with a minimum of 48 h between sessions [10]. Ultimately, patients in both groups were reassessed using the same tests as at baseline. The researcher responsible for conducting the assessments at baseline and at the end of the exercise program was blinded to patient allocation to the study groups. The platform used for the sessions was WhatsApp®, which is widely used among the population and is free. The exercise prescription was based on the same principles for healthy children and adults, including the duration and type of physical activity, as outlined by the *American College of Sports Medicine* [27]. The program aimed to improve muscle strength and functional capacity, tailored to each child individually.

At the initial assessment, participants and their guardians received a sealed brown envelope indicating the group to which the participant was allocated, according to the randomization. They were also informed that they would receive a text message from a researcher via the WhatsApp® application to schedule the first follow-up session. The researcher was responsible for initiating contact and maintaining follow-up with the patient throughout the 12 exercise sessions. In the event of unforeseen circumstances, participants were allowed to reschedule the session once more within the same week to manage it better. Standard outpatient medical care was maintained, with no differences between the groups. For adherence monitoring, participants were given a diary to record the date of each video call. The researcher responsible for the phone contact also kept a record of the activity using a standardized table that included the date, time, and duration.

The IG performed the exercises described in our study protocol [28] through pre-scheduled video calls conducted by a trained researcher twice a week. The protocol included warm-up, aerobic, and anaerobic exercises for upper and lower extremities — with or without resistance using elastic bands — and final stretches held for 20–30 s. For strength (anaerobic) training, the exercises were performed freely, against gravity, or using elastic bands (mini-bands) provided by the researchers, which were returned at the end of the study. Strength training for the upper limbs (UL) and lower limbs (LL) was performed without resistance (for children aged 6 to 12 years) or with resistance (for those aged 13 to 18 years) using elastic bands, with a gradual increase in intensity over three phases. The mini-bands of different colors were adjusted according to each child’s capacity. Sessions were coordinated to coincide with outpatient visits to minimize additional travel costs. Routine medications were maintained throughout and documented at baseline. Both groups were reassessed at the end of the intervention.

Patients in the control group (CG) also received a video call, during which they were educated about the importance of exercise. They received a non-mandatory request to perform basic breathing exercises (deep breathing or three times inspiration). Routine consultations, medication regimens, and laboratory tests were maintained according to the Nephrology service's established routine. To ensure adherence, all participants were provided with a diary to record the date of each video call. The researcher responsible for the phone contact also documented the activity using a standardized table, including the activity's date, time, and duration. Furthermore, adverse events were monitored during each session to assess the program's safety in both groups. Patients were monitored during all the video calls and questioned about possible adverse events during the activities. They were asked about dyspnea, excessive discomfort, muscle discomfort, and other symptoms. In addition, they were monitored with a portable pulse oximeter to check their heart rate (HR) and oxygen saturation.

### Statistical analysis

A total sample size of 17 patients was required to detect an effect size of f = 0.25, with 80% power and a significance level of 5%, in two groups and two evaluations. Adding 15% for possible follow-up losses, 19 patients were required per group. The sample size was calculated using G*Power 3.1.9.7.

The data were expressed as absolute and relative frequencies, mean and standard deviation when symmetrical, or median and interquartile range (IQR) when asymmetrical. Normality was assessed using the Shapiro–Wilk test. Groups were compared using the Chi-square, Fisher's Exact, Student's t-test, and Mann–Whitney tests. Due to follow-up losses, generalized estimating equations (GEE) models were used to analyze parameter changes, considering the main effects of group and time, and the group*time interaction, with Sidak's test for multiple comparisons. The results were presented as means and 95% confidence intervals (CI). The significance level was set at 0.05. Analyses were performed using the *Statistical Package for Social Sciences* (SPSS) version 25.

## Results

### Patient characteristics

**Fig. 2:**
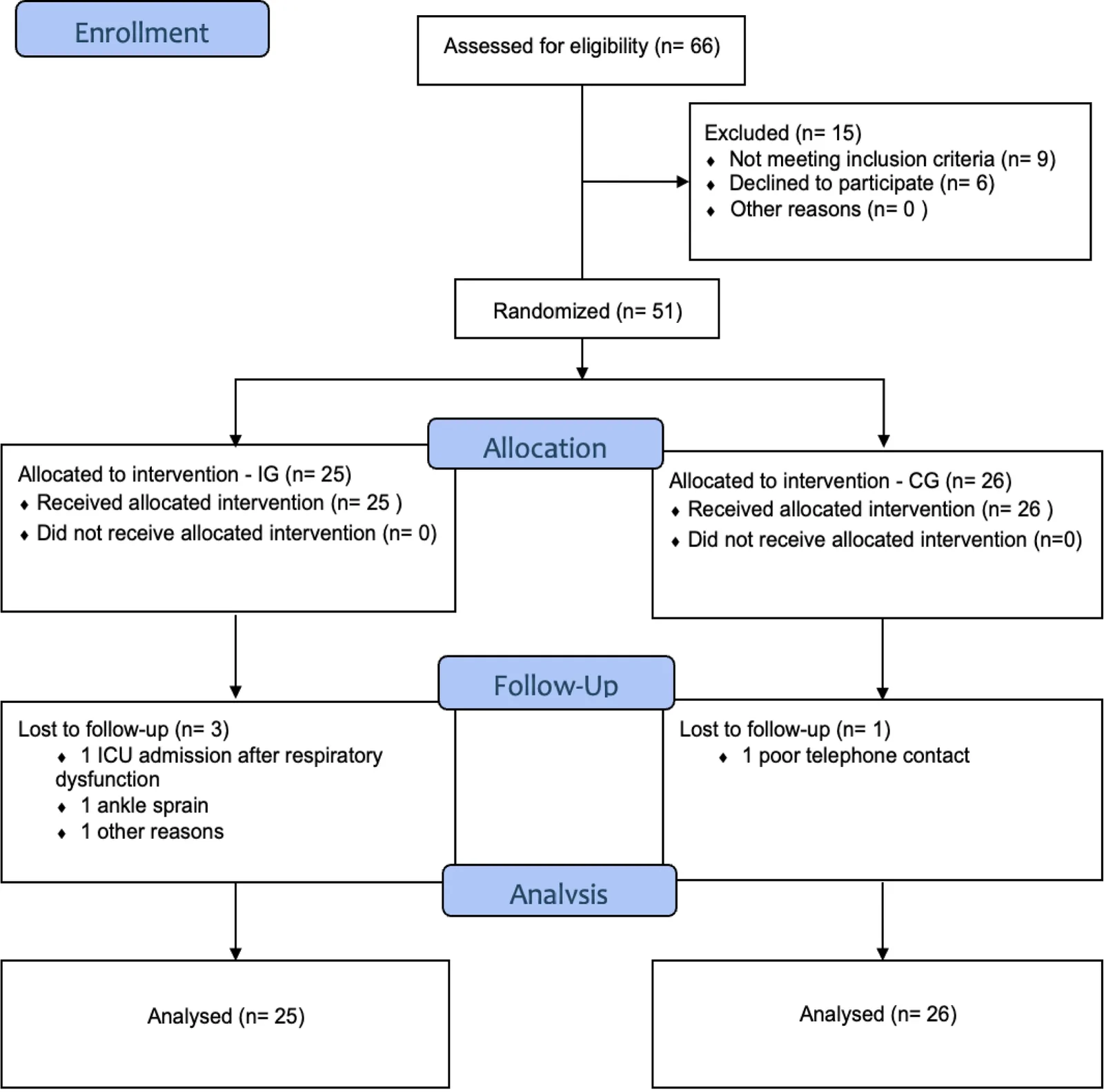
Flow diagram of the trial

Of 66 potentially eligible patients, 15 did not fulfill the inclusion criteria (Fig. 2). We also evaluated the profiles of the 15 patients not included in this study. Nine did not meet the inclusion criteria (3 cardiopathy, 2 severe neuromotor developmental delays, 1 cerebral palsy, 1 myelomeningocele, 1 suture dehiscence with difficult healing, and 1 acute rejection requiring dialysis). The remaining six patients declined participation. Therefore, 51 patients were randomized to either the IG (*n* = 25) or the CG (*n* = 26). There were no significant differences in age (p = 0.061), gender (p = 0.186), eGFR (p = 0.930), or creatinine values (p = 0.158) when compared to the 51 patients who participated in the study. During the follow-up, four patients dropped out of training after the initial evaluation; however, they were not excluded from the final data analysis.

The most prevalent diseases in IG and CG were, respectively, urinary tract malformations (kidney dysplasia 20% and 26.9%, p = 0.801; autosomal dominant polycystic kidney disease 16% and 7.7%, p = 0.419; posterior urethral valves 4% and 7.7%, p = 1.000; vesicoureteral reflux 4% and 3.8%, p = 1.000; ureteropelvic junction stenosis 4% and 3.8%, p = 1.000), glomerular diseases (steroid-resistant nephrotic syndrome 8% and 11.5%, p = 1.000; chronic glomerulopathies 4% and 0%, p = 0.490; focal segmental glomerulosclerosis 0% and 7.7%, p = 0.490) and unknown causes 24% and 7.7%, p = 0.140.

Patients from both groups received similar medication and maintained the same medical regimen throughout the study. Other group characterization data are described in Table 1. In addition, values of functional capacity and peripheral muscle strength were similar between groups at baseline At baseline, demographic and clinical characteristics were similar for both groups, i.e., systolic blood pressure (SBP) (p = 0.056), diastolic blood pressure (DBP) (p = 0.060), and heart rate (HR) (p = 0.937). Compliance with the telerehabilitation was very good at 10.2 ± 3.3 days (85%) and 11.2 ± 2.4 days (93.3%) for the IG and CG, respectively (p = 0.210). No adverse effects or complications related to training were reported in both groups.

**Table 1:** Demographics and clinical characteristics at baseline

	IG (*n* = 25)	CG (*n* = 26)	p-value
Age (years)	11.8 ± 3.5	11 ± 3.6	0.800
Gender (Male)	13 (52.0)	9 (34.6)	0.332
Z score (BMI/age)			
Eutrophy	20 (80.0)	19 (73.1)	0.776
Overweight	3 (12.0)	5 (19.2)	
Obesity	2 (8.0)	2 (7.7)	
Z score (height/age)			
Very short height	3 (12.0)	4 (15.4)	0.112
Short height	6 (24.0)	1 (3.8)	
Adequate height	16 (64.0)	21 (80.8)	
Time after Tx (months)	2 [1–7]	3 [1–8]	0.977
Days in ICU	5 [4–6]	6 [5–7]	0.073
Donor type			
Deceased donor	22 (88.0)	22 (84.6)	1.000
Living donor	3 (22.0)	4 (15.4)	
Patients on Hemodialysis before Tx	13 (52.0)	7 (26.9)	0.122
Time on hemodialysis (months)	7 [3–10]	6 [5–18]	0.817
Patients on PD before Tx	9 (36.0)	15 (57.7)	0.204
Time on PD (months)	12 [3–17]	7 [4–24]	0.953
eGFR^a^ (ml/min/1.73 m^2^)			0.936
≥ 90	10 (40.0)	9 (34.6)	
60–89	9 (36.0)	10 (38.5)	
30–59	5 (20.0)	5 (19.2)	
< 30	1 (4.0)	2 (7.7)	
Physical activity beforeTx^b^			
Yes	15 (60.0)	11 (42.3)	0.325
No	10 (40.0)	15 (57.7)	
Drugs			
FK506	21 (84.0)	23 (88.5)	0.703
CsA	5 (20.0)	3 (11.5)	0.465
Mycophenolate sodium	22 (88.0)	21 (80.8)	0.703
Prednisone	23 (92.0)	26 (100)	0.235
Amlodipine	7 (28).0	7 (26.9)	1.000
Others	25 (100)	24 (92.3)	0.490
Associated comorbidities			
None	14 (56.0)	15 (57.7)	1.000
SAH	4 (16.0)	7 (26.9)	0.543
DM	5 (20.0)	1 (3.8)	0.099
Others	3 (12.0)	6 (23.1)	0.465

Data are mean ± standard deviation, n (%), or median [IQR]

*IG*: intervention group; *CG*: control group; *BMI*: body mass index; *Tx*: transplantation; *ICU*: intensive care unit; *PD*: peritoneal dialysis; *eGFR*: estimated glomerular filtration rate; *FK506*: tacrolimus; *CsA*: ciclosporin; *SAH*: systemic arterial hypertension; *DM*: diabetes mellitus

^a^Estimated glomerular filtration rate calculated by the Schwartz formula

^b^Physical activity: activities performed at least three times a week, for at least 30 min a day

**Table 2:** Results of tests on functional capacity, peripheral muscle strength, quality of life, and laboratory measures before and after training of patients randomized to IG or CG

	IG (*n* = 25)		CG (*n* = 26)		p-value	
	Before	After	Before	After	Factor time	Time × group interaction
Systolic blood pressure (mmHg)	118.3 (112.9–123.8)	113.9 (107.7–120.7)*	110.2 (104.4–116.0)	106.4 (99.7–113.1)*	0.050	0.866
Diastolic blood pressure (mmHg)	83.6 (78.8–88.3)	77.7 (72.1–83.3)	76.7 (71.7–81.6)	73.4 (69.2–77.6)	0.021	0.507
Score Z (BMI)	0.16 (−0.26–0.58)	0.32 (−0.11–0.76)	0.29 (−0.16–0.74)	0.42 (−0.02–0.86)	0.028	0.788
MSWT Distance (m)	314.0 (252.8–375.2)	404.6 (337.3–471.9) *¥	316.9 (266.5–367.4)	317.7 (267–368.4)*	0.000	0.000
MSWT Predict (m)	1015.7 (945.0–1086.3)	1011.1 (944.3–1077.9)	949.8 (875.8–1023.7)	933.5 (868.5–998.4)	0.253	0.519
MSWT % of predict	30.4 (25.5–35.2)	39.4 (34.2–44.7) *¥	34.6 (29.1–40)	34.9 (29.4–40.4)*	0.000	0.000
Handgrip (Kg)						
RUL	15.6 (12–19.1)	16.3 (12.9–19.8)*	13.6 (10.5–16.6)	13.9 (10.6–17.3)*	0.015	0.408
LUL	13.3 (10.4–16.2)	14.3 (11.5–17.2)*	11.5 (8.9–14.2)	11.8 (8.9–14.8)*	0.050	0.284
DUL	15.3 (11.4–19.2)	16.2 (12.4–19.9)*	13.7 (10.6–16.9)	14.1 (10.6–17.6)*	0.012	0.369
*PedsQL™*						
Physical	72.6 (67.8–77.4)	78.6 (73.8–83.4)*	75.5 (69.1–81.8)	79.4 (74.8–84.1)*	0.008	0.596
Emotional	70.2 (63.2–77.2)	73.4 (67.6–79.1)	65.4 (57.2–73.5)	72.9 (65.2–80.6)	0.063	0.452
Social	76.8 (70.0–83.6)	82.7 (78.6–86.9)*	77.9 (70.9–84.9)	84.1 (78.2–89.9)*	0.004	0.945
School Functioning	58.2 (50.7–65.7)	62.8 (56.0–69.7)	65.6 (61.2–69.9)	65.9 (59.8–72)	0.273	0.335
Overall	69.9 (65.5–74.3)	75 (71.3–78.6)*	71.7 (67.6–75.7)	76.1 (71.9–80.3)*	0.001	0.814
Laboratory measures						
eGFR ml (min^−1^ 1.73 m^2^)	82.6 (73.2–92)	83.2 (74.2–92.2)	76.8 (66.1–87.5)	71.3 (61.2–81.3)	0.409	0.305
Hemoglobin (g/dL)	12.6 (12–13.2)	12.6 (12–13.2)	11.7 (11.2–12.3)	12.1 (11.6–12.6)	0.469	0.371
Hematocrit (%)	37.3 (35.6–39.1)	36.5 (34.7–38.3)	34.8 (33.1–36.5)	35.6 (34.2–37)	0.966	0.211
Urea (mg/dL)	35.7 (30.5–40.9)	38.8 (34.8–42.8)	44.5 (38–51)	47.3 (39–55.7)	0.221	0.954
Creatinine (mg/dL)	0.8 (0.6–0.9)	0.7 (0.6–0.9)	0.9 (0.7–1.1)	0.9 (0.8–1.1)	0.871	0.546
Potassium (mEq dl)	4.5 (4.3–4.7)	4.4 (4.3–4.6)	6 (3–9)	4.4 (4.3–4.6)	0.297	0.315
Magnesium (mg/dL)	1.6 (1.5–1.7)	1.9 (1.4–2.4)	1.8 (1.7–1.9)	1.8 (1.7–2)	0.217	0.385
Inorganic phosphate (mg/dL)	4.8 (4.5–5.1)	4.8 (4.4–5.2)	4.7 (4.3–5)	4.8 (4.5–5.2)	0.589	0.472

*IG*: intervention group; *CG*: control group; *BMI*: Body Mass Index; *MSWT*: Modified Shuttle Walk Test; *RUL*: right upper limb; *LUL*: left upper limb, *DUL*: dominant upper limb; *eGFR*: estimated glomerular filtration rate. *PedsQL™*: Pediatric Quality of Life Inventory

^*^differs significantly from PRE within the group (significant time effect); ¥ differs significantly from CG at the same time point (significant interaction effect)

Regarding the time of recruitment after KT, we had in the IG 18 (72%) with ≤ 6 months and 7 (28%) > 6 months. In the CG, 17 (65.4%) patients had ≤ 6 months and 9 (34.6%) > 6 months (p = 0.836). We analyzed the patients before and after 6 months of transplantation. The changes observed, when present, in the SWT (p = 0.328), peripheral muscle strength (p = 0.654), total QoL (0.245), BMI score Z (p = 0.982), SBP (p = 0.738), or DBP (p = 0.821) were independent of the time since transplantation.

### Capacity functional test

Table 2 shows data on functional capacity test before and after training periods in both groups. There was a significant increase in the distance covered in MSWT in IG compared to CG (p = 0.000). The same pattern was observed for the predicted distance covered in the MSWT, with a significant increase in the IG (p = 0.000). After training, we observed no significant differences between groups regarding HR (p = 0.877). The values of SBP (p = 0.050) and DBP (p = 0.021) decreased after exercise training, regardless of group.

### Peripheral muscle strength

The peripheral muscle strength increased in both groups compared to baseline for right upper limb (RUL) (p = 0.015), left upper limb (LUL) (p = 0.050), and dominant upper limb (DUL) (p = 0.012). However, no significant increase was observed regarding the intervention (Table 2). The achieved values of the predicted percentage before and after the intervention were, respectively, 60.2% and 64.7% in IG and 59.3% and 59.8% in CG for RUL (p = 0.115), 57.5% and 62.7% in IG and 55.2% and 55.4% in CG for LUL (p = 0119), and finally, 61.1% and 65.9% in IG and 60% and 60.5% in CG for DUL (p = 0.131).

### Quality of life and laboratory measures

QoL improved across all PedsQL domains; however, a significant increase was observed only in the Physical, Social, and Overall domains (time effect only), with no effect of the intervention. The IG improved from a fair to a good rating in the overall score assessment, while the CG maintained a good rating in both assessments. Laboratory measures at baseline and after exercise training were similar in both groups (Table 2).

## Discussion

This randomized clinical trial, based on a telerehabilitation exercise program conducted over six weeks in pediatric patients after recent KT, demonstrated beneficial effects on functional capacity, muscle strength, and self-reported QoL. To the best of our knowledge, this is the first study to provide evidence of the beneficial effects of telerehabilitation exercise in children and adolescents following KT.

Patients with CKD, even after KT, experience a significant reduction in exercise capacity secondary to neuromuscular, metabolic, and cardiopulmonary disturbances induced by the disease, excess body volume, medication use, and anemia [8, 29]. Bonzel et al. [30] observed a significant decrease in maximal oxygen consumption (VO_2_ max) and maximal workload (W max) in transplanted children in comparison with healthy individuals. These results indicate that even after KT, transplanted children experience impairment in their exercise capacity. Supporting these findings, Painter et al. [9] assessed 25 children after KT and 15 on dialysis (nine underwent transplantation during the study and were re-evaluated). The study did not identify a significant improvement in pre- and post-transplant outcomes among the nine re-evaluated individuals, except for a significant increase in body fat percentage, which negatively impacted VO_2_ max. All study participants were physically inactive, with less than 10% of their time outside school dedicated to physical activity. The authors concluded that the children exhibited reduced exercise capacity, were physically inactive, and tended to gain weight after KT.

Our study corroborates these findings. The distance covered in the MSWT at baseline was below the predicted values for this population, with only 30.4% and 34.6% of the predicted values achieved in the IG and CG, respectively, demonstrating low functional capacity. After the training, this value significantly increased in the IG, reaching 39.4%, and there was no difference in the CG; however, it remained below the expected values. Physical inactivity leads to various health issues, including musculoskeletal changes, somatic conditions, overweight and obesity, circulatory problems, and even premature death [31–34]. Exercise provides significant health benefits, improving the function of several organs, increasing mental well-being, enhancing overall physical performance, and reducing body mass and mortality [35, 36]. The benefits of physical activity extend to all children, including those with special health needs [38], such as CKD and KT [37]. The study by Kohlmeier et al. [38] documents a considerable lack of physical activity among young KT recipients, and that engaging in physical activity was positively associated with cardiovascular health parameters.

In our study, a telerehabilitation-based exercise program resulted in a statistically significant increase in distance covered during the MSWT. The exercise protocol was based on the *American College of Sports Medicine* guidelines [27]. However, to ensure the safety of a remotely supervised program and to respect the limitations of patients who underwent recent transplantation, we divided the groups by age and assigned distinct activities. The younger group, aged 6 to 12 years, performed multicomponent exercises with gradual increases in repetitions; the older group, aged 13 to 18 years, performed a more conservative workout with light aerobic activity, strength exercises, progressive elastic resistance, and stretching [28]. These adaptations are supported by the *Physical Activity Guidelines for Americans* [39], which suggest that a healthcare professional should supervise children with functional limitations to assess their needs and adapt activities, as well as their duration and frequency, to make them as active as possible. Janaudis-Ferreira et al. [40] recently emphasized the importance of exercise training for pediatric patients before and after solid organ transplants and highlighted the necessity of individualized exercise programs that include aerobic, resistance, and flexibility activities tailored to each child's clinical condition, age, and individual characteristics.

Our telerehabilitation follow-up showed significant results in functional capacity, even though the patients could not reach normal values. In the intragroup assessment, the average increase in distance covered during the MSWT was 90.6 m in the IG from pre- to post-training, compared to only 0.8 m in the CG. Furthermore, when comparing the groups, the IG showed an average increase of 86.9 m in distance covered compared to the CG, which was statistically significant. This finding supports the study by Abd-Elmonem et al. [10] on progressive resistance exercise in children with CKD. The exercise group showed a significant improvement in functional capacity (6MWT), whereas the standard medical care group showed no significant change. It is important to highlight that, in our study, the patients exhibited significant physical deconditioning at baseline. Despite the significant improvement in the MSWT, the patients in the IG maintained values well below the predicted distance. The short duration of the training program and the limited number of aerobic exercises may have influenced these findings.

Among healthy children and adolescents, exercise can improve physical health and promote mental health, reducing anxiety and improving academic performance, body image, and mood [36]. Our study found a significant increase in PedsQL domains in both groups between pre- and post-assessments, but there were no significant differences in the intervention. This finding contrasts with those reported by Abd-Elmonem et al. [10] regarding QoL, in which, after resistance exercise training, they found a significant increase in the PedsQL score in the intervention group and deterioration across all domains in the CG. Classifying the findings, we observed that although no significant relationship was found between the intervention, the IG improved its rating from fair to good in the physical domain and in the overall score. At the same time, the CG maintained a good rating in both assessments. Although no significant relationship was found, the increase observed in both groups compared to baseline is due to telemonitoring, which provides personalized, weekly follow-up and the opportunity to address questions, interact, and motivate this population, thereby improving QoL domains.

Regarding peripheral muscle strength, measured by handgrip, we observed values below the predicted percentage in the dominant limb for both the IG (61.1%) and the GC (60%). This finding is consistent with the study by Frantzeski et al. [41], in patients after KT (78.8% of the predicted values). Furthermore, after our program exercise, this variable increased in both groups (slightly more in the IG), regardless of the intervention. This result aligns with Goldstein and Montgomery's pilot study [42], which found no significant effect on handgrip strength (p = 0.07) but improvements in eight out of ten children with CKD after three months of exercise. We hypothesize that the relatively short follow-up period may have influenced our findings. Additionally, most of our sample consisted of children under 13 years of age (13 in the IG and 18 in the CG), and no increase in exercise load was incorporated during the intervention for this age group.

Telerehabilitation is a promising tool for remotely monitoring patients and can be an ally in responding to health emergencies and providing access to pediatric care in remote and underserved areas [12]. A longitudinal study [43] in adults post-KT evaluated a 1-year personalized exercise program with telehealth monitoring for adherence, demonstrating high adherence and highlighting the benefits of exercise for physical fitness and cardiovascular health, as well as the role of telehealth in supporting long-term adherence. A recent literature review [16] on patients after KT highlighted the benefits of this therapeutic modality, such as reduced travel time, lower costs, improved adherence, and increased self-sufficiency. The authors recommend implementing this technology as standard of care to provide patients with high-quality care at home. Our results demonstrate the effectiveness of this resource in monitoring these patients, particularly in physical aspects. We also emphasize the importance of telerehabilitation follow-up, even for the CG, which showed some improvement in functional capacity and peripheral muscle strength over the follow-up period, despite not engaging in multicomponent and strength exercises.

Simple interventions, such as early incremental physiotherapy and emphasizing physical activity at levels comparable to healthy peers, significantly impact cardiorespiratory fitness and mental health in children following KT [44]. Additionally, home-based training can be safe and effective for patients with CKD [45], and the telerehabilitation approach can be an additional tool for high-quality follow-up. A large portion of the sample (39.2%) was from other states in Brazil, yet adherence was satisfactory, supporting the success of telerehabilitation interventions for closer follow-up of these patients. No adverse effects were observed during the implementation.

This study has some limitations that should be considered in analyzing and interpreting the results. Barriers, such as limited digital infrastructure among some patients, may hinder the approach. Furthermore, a weather event in Porto Alegre in 2024 may have reduced patient recruitment and adherence.

In conclusion, this randomized clinical trial demonstrated that a short-term exercise program via telerehabilitation significantly improved pediatric patients' functional capacity after KT. Although no significant effect of telerehabilitation-based training on peripheral muscle strength and QoL was observed, substantial increases in these parameters were observed in both groups from baseline to the end of follow-up, especially in the IG. We believe telerehabilitation has great potential for pediatric kidney transplant recipients, especially because significant dysfunctions in functional capacity persist even after KT. Additionally, the approach was easy to implement, showed no adverse events, was safe, and had excellent adherence. Finally, the scarcity of studies on transplant patients, especially in the pediatric population after KT, makes the findings of this study particularly relevant for implementing exercise telehealth programs to improve functional capacity and peripheral muscle strength.

## Supplementary Information

Below is the link to the electronic supplementary material.

Graphical abstract (PPTX 81 KB)

## Data Availability

Datasets used and/or analyzed during the present study are available from the corresponding author on reasonable request.
