# Supplementary material for: Telerehabilitation exercise program in pediatric patients after kidney transplantation: a randomized clinical trial
Source: Pediatr Nephrol. 2026 Feb 4;41(7):2145–55. doi: 10.1007/s00467-025-07123-3 (PMC13197309; doi:10.1007/s00467-025-07123-3)
Supplement: Supplementary file 1 — Graphical abstract (PPTX 81 KB) [file 467_2025_7123_MOESM1_ESM.pptx]

## Slide 1
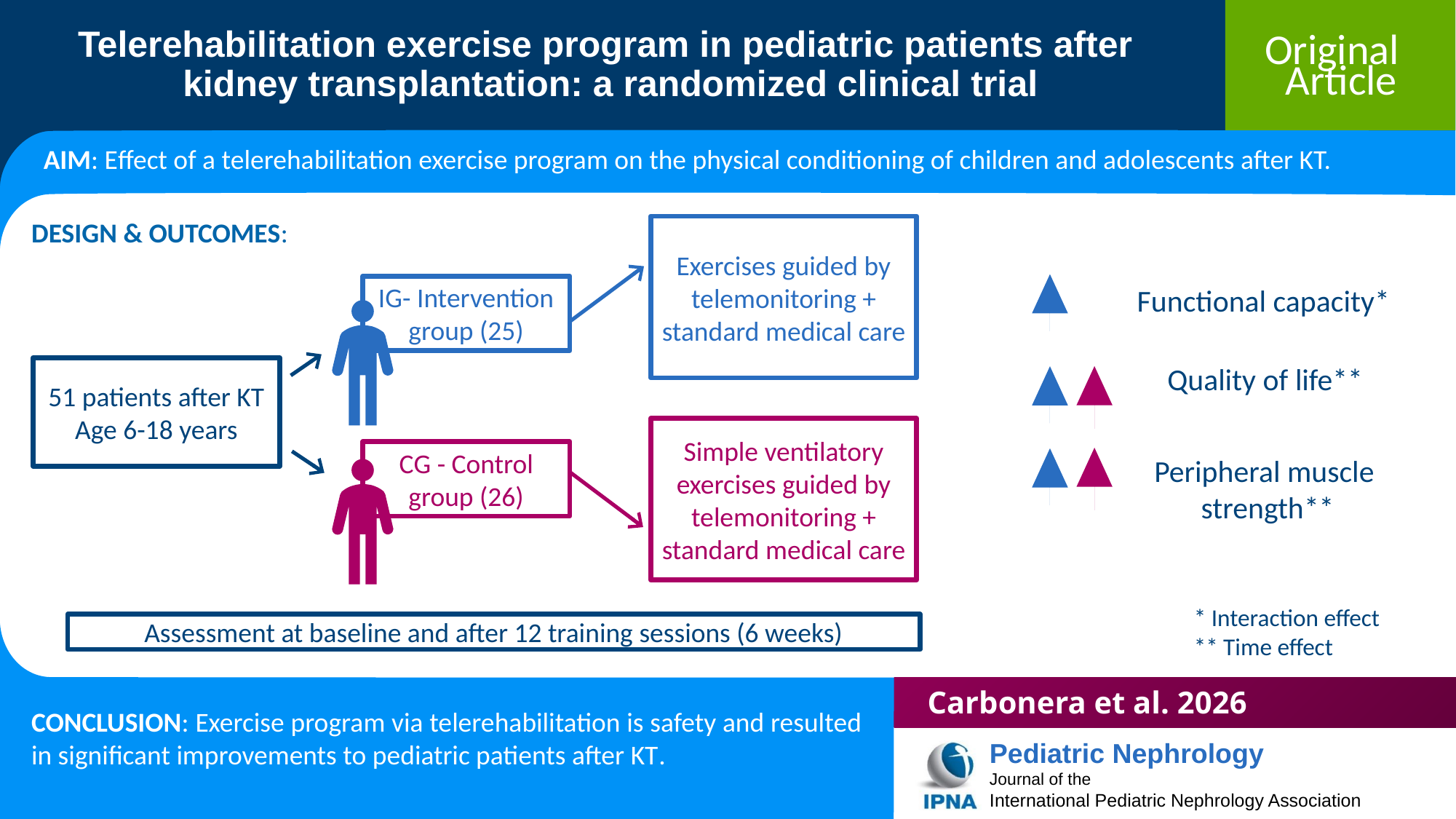

Telerehabilitation exercise program in pediatric patients after
kidney transplantation: a randomized clinical trial
AIM: Effect of a telerehabilitation exercise program on the physical conditioning of children and adolescents after KT.
DESIGN & OUTCOMES:
Exercises guided by telemonitoring + standard medical care
Functional capacity*
IG- Intervention group (25)
Quality of life**
51 patients after KT
Age 6-18 years
Simple ventilatory exercises guided by telemonitoring + standard medical care
CG - Control group (26)
Peripheral muscle
strength**
* Interaction effect
** Time effect
Assessment at baseline and after 12 training sessions (6 weeks)
Carbonera et al. 2026
CONCLUSION: Exercise program via telerehabilitation is safety and resulted in significant improvements to pediatric patients after KT.
